# True Success in Dentistry

**Published:** 1907-08

**Authors:** Carl P. Norris

**Affiliations:** Lillington, N. C.


### TRUE SUCCESS IN DENTISTRY.

By Dr. Carl P. Norris, Lillington, N. C.

With the present day's rush of young men into every kind of business for the sake of the money to be obtained therefrom, it may be well for the dental profession to pause a moment to consider who are to be the future standard-bearers of their high calling. When we look around us and see missing from our ranks many strong men, who but a short time ago occupied prominent and influential positions in our society and profession, we sometimes wonder, will not the lucrative allurements of business life deprive dentistry of men who could and would otherwise raise its standard higher in the estimation of the people and will not this turning of the tide cause incompetent men to force their way into the ranks, solely for the sake of their own selves, and thus cause a blot upon the profession? But what is the remedy for this? Will charging higher prices change the course of affairs and alleviate the evil tendency? No. We must strike at a deeper and more vital point. We must first teach young men what true success really is.

Were we to inquire of different persons the meaning of the word, each would give a different answer; one would say it is accumulation of money, another religious zeal, still another will say, it is education in science, art or literature; another, possibly, that cheerfulness and enthusiasm constitute true success.

All of these are partially correct, but it is safer and better to use a broader term, one that will strike at the heart of the word. We can safely say, without being contradicted, that true success in dentistry, as well as in any other profession, or in any business, is the fulfillment of our desires and aims, provided, however, that the fulfillment is a benefit both to ourselves and to those with whom we come in contact. In order to attain this success many duties devolve upon the practitioner. Of the many we desire to briefly enumerate a few.

The duty we owe to our society. We cannot be too loyal to it. We should be proud of it and honor it by our presence, it has been a nucleus of education from which has radiated honor, distinction and beneficence. It is through these associations that we have efficient and ethical members of our state boards and these boards have stimulated the dental colleges to a higher ideal, and have run the purchasing diploma concerns out of business. We should look forward to our annual meeting with pleasure, as a great Summer Dental School where we exchange methods and ideas freely.

I am exceedingly sorry to say that there are many good dentists in our Old North State who are not members of this organization. This society, to fulfil its objects needs the support of every ethical dentist in the state and it is our duty to work toward bringing more of them, and if possible, every one of them into it. Let us never become offended simply for the reason that some of its proceedings do not exactly conform to our individual wishes.

Disloyalty on the part of members of the society is neither becoming nor does it reflect credit upon them. If we are wise enough to perceive the short-comings of the convention, let us give better proof of our ability by taking a hand and helping to make it what we think it ought to be.

Every truly ethical dentist is duty bound to his professional brother and should be forever on his guard, using every artful tact to shield his reputation, whenever his skill or the honesty of his purpose has been questioned or assailed by an irate patient. We must at all times bear in mind, and that it matters not how unskilled a piece of work coming under our supervision may appear from its present condition, at sometime during our career, we have had the misfortune to send from our hands, and our offices, work that was equally as bad if not worse.

Therefore, remember, that if we fail to observe our ethics and through thoughtlessness, malice, aforethought or otherwise, criticise our brother harshly the time will surely come when the same opportunity will come to him and the science of ethics and the milk of human kindness must be indeed deeply rooted in his soul if he declines to avail himself of this splendid opportunity.

It is our bounden duty to keep strict guard over our feelings and be very sure to mediate carefully and conscientiously, lest some petty professional jelousy—some lurking brutality—should cause some concealed criticism to find lodgment in our breast, against the success of our brother. Too often it is the case. With Burns we mourn,

"That man whose Heaven erected face
The smiles of love adorn,
Man's inhumanity to man
Makes countless thousands mourn."

We should always be mindful of our duty to our patients. Study their needs. Be honest, candid and attentive. Everything should be done to convince our patients that the care of the oral cavity is absolutely necessary to the conservation of health.

Civilized people remove the accumulations of waste and foreign debris from other portions of the body but often neglect so important a part as the mouth.

It is our duty to place the mouth in aseptic condition by our operative skill and mechanism, aided by such antiseptics and germicides as our knowledge of therapeutics and bacteriology may suggest—instructing the patient as to the future care of the teeth.

We should always strive to do our best when performing any service, be it ever so small. It is said that our character is written in our work.

Dentists can and ought to be greater benefactors, when we note the fact that fifty per cent. of all diseases are directly traceable to defective teeth, or a failure to rightly use them and by noting that ninety-five per cent. of the entire population have defective teeth, we readily see that the public offers the dentist abundant opportunity to become its benefactor in helping to fortify his brother against disease and thereby lengthen the period of human life.

Our example before the world should be such as to win for us respect and influence in our community. And never let us act in such manner as would tend to bring reproach upon ourselves and the honorable profession which we represent.

Each individual is engraving his own monument. Let each one live a life worthy of praise, and our monument will be unveiled with a clear inscription of the power we have maintained.

"The sweetest lives of those to duty wed,
Whose deeds both great and small,
Are closeknit strands of an unbroken thread
Where love ennobles all.
The world may sound no trumpets, ring no bells,
The Book of Life the shining record tells."

